# Serological and molecular screening for zoonotic pathogens among wild hedgehogs (*Erinaceus europaeus*) from urban areas of Poland

**DOI:** 10.2478/jvetres-2025-0022

**Published:** 2025-04-04

**Authors:** Hanna Turlewicz-Podbielska, Jakub Jędrzej Ruszkowski, Maria Pisarek, Łukasz Adaszek, Małgorzata Pomorska-Mól

**Affiliations:** 1 Department of Preclinical Sciences and Infectious Diseases, Faculty of Veterinary Medicine and Animals Sciences, Poznań University of Life Sciences, 60-637 Poznań, Poland; 2 Department of Animal Anatomy, Faculty of Veterinary Medicine and Animals Sciences, Poznań University of Life Sciences, 60-625 Poznań, Poland; 3 Department of Epizootiology and Clinic of Infectious Diseases, Faculty of Veterinary Medicine, University of Life Sciences in Lublin, 20-612 Lublin, Poland

**Keywords:** *Anaplasma* spp., hedgehogs, *Rickettsia* spp., synurbic species, zoonotic pathogens

## Abstract

**Introduction:**

Wild European hedgehogs (*Erinaceus europaeus*) can carry various pathogens potentially harmful to humans. This study was conducted to determine the occurrence of selected zoonotic pathogens in European hedgehogs from urban areas of central-western Poland.

**Material and Methods:**

Sixty-nine samples (43 of sera and 26 spleens) were collected from 54 hedgehogs brought to the Wildlife Rehabilitation Centre in Poznań, Poland, between June 2020 and September 2023. Antibodies against *Coxiella burnetii*, hepatitis E virus genotype 3, *Toxoplasma gondii* and *Trichinella* spp. in serum samples were determined using commercial ELISA tests. A PCR was used to evaluate the prevalence of *Anaplasma* spp., *Ehrlichia* spp., *Borrelia* spp., *Rickettsia* spp. and *Leptospira* spp. genetic material in spleens.

**Results:**

The genetic material of *Anaplasma phagocytophilum* was found in 18 out of 26 spleens (69.23%; 95% confidence interval (CI): 50.01–83.50), and the genetic material of *Rickettsia helvetica* in 4 out of these 26 (15.38 %; 95% CI: 6.15–33.53). All *Rickettsia*-positive spleens were also positive for *Anaplasma* spp. None of the other pathogens or antibodies against them were detected.

**Conclusion:**

This study provides valuable insights into the prevalence of some zoonotic pathogens in urban hedgehog populations and their potential impact on public health and urban biodiversity.

## Introduction

European hedgehogs (*Erinaceus europaeus*) are small, nocturnal insectivorous mammals spread widely across Europe, Asia and Africa (31). The growing presence of hedgehogs in cities inevitably brings them into contact with humans directly (*e.g*., when people transport injured hedgehogs to wildlife rehabilitation centres) and indirectly (such as through feeding bowls in gardens or through pets). Hedgehogs have a varied diet, including earthworms, slugs, insects, small rodents, amphibians, lizards and birds, eggs and even pet food (3), and as opportunistic feeders their search for food often brings them into close contact with people. These animals can carry various pathogens which are potentially harmful to humans (31). Pathogen transmission occurs mainly through contact with infected animals or their excreta (23). Monitoring infectious diseases in hedgehogs is important in order for threats to public health and the coexistence of urban ecosystems to be recognised, in line with the One Health approach.

This study aimed to assess the occurrence of selected zoonotic pathogens in wild hedgehogs from central-western Poland. Commercial multispecies ELISAs were used to determine the seroprevalence of *Coxiella burnetii*, hepatitis E virus genotype 3 (HEV-3), *Toxoplasma gondii* and *Trichinella* spp. in serum from European hedgehogs. A PCR was employed to evaluate the prevalence of *Anaplasma* spp., *Ehrlichia* spp., *Borrelia* spp., *Rickettsia* spp. and *Leptospira* spp. genetic material in hedgehogs’ spleens. The importance of the results of the study extends beyond what it indicates about the ecosystem around urban hedgehogs, because all the pathogens screened for are considered pathogenic to humans (3, 8, 31).

## Material and Methods

### Sample collection

Because hedgehogs are protected by Polish law with the Regulation of the Minister of the Environment of 16 December 2016 on the protection of animal species and its amendment (14, 15), all procedures were approved under the appropriate regulations and carried out with permit No. WPN-II.6401.366.2020.TE issued by the Regional Directorate for Environmental Protection in Poznań, Poland.

Sixty-nine samples (43 of sera and 26 spleens) were obtained from 54 hedgehogs brought to the Wildlife Rehabilitation Centre (WRC) in Poznań between June 2020 and September 2023 after being found incurably sick, fatally injured or too young to survive on their own. All hedgehogs were found in the central-western Polish city of Poznań (16°55′ E; 52°25′ N) in the Wielkopolskie Voivodeship. When admitted to the WRC, the hedgehogs were kept in isolation until sample collection (within 12 h of admission) to reduce the possibility of nosocomial infections. After collection, blood samples were centrifuged to obtain serum. In addition, spleen samples from 26 hedgehogs that were dead on arrival or were euthanised at the WRC for ethical reasons (if the animal had a spinal or bone fracture) were collected. Euthanasia was with xylazine and ketamine administered intramuscularly and next pentobarbital administered intravenously. Serum and spleen samples were collected from 15 animals, blood only was taken from 28 animals, and only spleen samples were removed from 11 animals. Sera and spleens were stored at –70°C until analyses. The following information for each hedgehog was recorded: delivery date, sex, body weight and health status. Detailed information about the structure of the sampled population is presented in Table 1 (ELISAs) and Table 2 (PCR).

**Table 1.** The structure of the hedgehog population tested for seroprevalence

Variable		Number of individuals
Health status	Polytrauma	29
	Respiratory disease	1
	Gastrointestinal disease	1
	Others (cachexia)	4
	Healthy	4
	No data	4
Sex	Male	27
	Female	16
Body weight (g)	<400	10
	400–650	16
	651–900	13
	>900	4
Total		43

**Table 2.** Results of hedgehog spleen sample testing in PCR, indicating the number of positive individuals and prevalence of *Anaplasma* spp. and *Rickettsia* spp. among the population subgroups

Variable		Total	*Anaplasma* spp.				*Rickettsia* spp.			
			+	Prevalence (%)	95% CI^a^	P-value	+	Prevalence (%)	95% CI^a^	P-value
Health status	Polytrauma	15	10	66.67	41.71–84.82	0.046	4	26.67	10.90–51.95	0.325
	Respiratory disease	2	0	0.00	0.00–65.76		0	0.00	0.00–65.76	
	Gastrointestinal disease	2	1	50.00	9.45–90.55		0	0.00	0.00–65.76	
	Others (cachexia)	7	7	100	64.57–100		0	0.00	0.00–35.43	
Sex	Male	13	8	61.64	35.52–82.29	0.671	3	23.08	8.18–50.26	0.587
	Female	13	10	76.92	49.74–91.82		1	7.69	1.37–33.31	
Body weight (g)	<400	7	5	71.43	35.89–91.78	0.980	0	0.0	0.00–35.43	0.399
	400–650	12	8	66.67	39.06–86.19		3	25.00	8.89–53.23	
	651–900	3	2	66.67	20.77–93.85		0	0,00	0.00–56.15	
	>900	4	3	75.00	30.06–94.44		1	25.00	4.56–69.94	
Total		26	18	69.23	50.01–83.50		4	15.38	6.15–33.53	

a – lower and upper values for the 95% confidence interval (CI); + – positive for the given pathogen

### Screening for antibodies against *T. gondii*, HEV-3, *C. burnetii* and *Trichinella* spp. in serum samples

A commercial ELISA (Toxoplasmosis Indirect Multi-species; IDVet, Grabels, France) was used to detect anti-*Toxoplasma* IgG antibodies according to the manufacturer’s instructions. Antibodies to *C. burnetii* were detected by a commercial ELISA test using microtitre plates pre-coated with the *C. burnetii* phase I and II strains (Q Fever Indirect Multi-Species; IDVet).

The ratio of the sample OD (optical density) to the mean OD of the kit-component positive control (signal-to-positive ratio – S/P) was calculated by also incorporating a read of the kit-component negative control using the following equation: S/P = (OD_sample_ – OD_negative_ control)/(OD_positive_ control – OD_negative_ control). Samples with S/P ≥ 0.5 were considered positive and samples with S/P ≤ 0.4 were considered negative. Samples with S/P ratio > 0.4 and <0.5 were considered doubtful.

Anti-HEV-3 IgG antibodies were determined using the Hepatitis E Indirect Multi-species ELISA (IDvet) according to the manufacturer’s instructions and retaining the positive and negative controls provided. It is a duplicate-well test, where even-numbered wells are coated with a recombinant antigen from the capsid of HEV-3, and odd-numbered wells are uncoated. The cut-off values (S/P%) that allowed the sample to be considered positive, doubtful or negative were calculated using the following formula: (OD_sample_/OD_positive control_) × 100%. The serum was considered positive when its cut-off value exceeded the borderline seropositivity of 70%. A doubtful result ranged from 60% to 70%, and OD values were below 60% for negative sera.

Seroprevalence of *Trichinella* spp. was assessed using an indirect multi-species ELISA for trichinellosis (IDvet). The OD values obtained were used to determine the S/P% for each of the test samples using the following formula: S/P (%) = (OD_sample_ – OD_negative control_)/(OD_positive control_ – OD_negative control_) × 100. No additional positive or negative controls were used besides those supplied in the kit. Samples with an S/P% less than 50 were regarded as negative, an S/P% between 50 and 60 was regarded as doubtful and the test was considered positive if the S/P% was greater than or equal to 60.

The ODs of all ELISA results were read at 450 nm using an Infinite 200 PRO microplate reader (Tecan Group, Männedorf, Switzerland) immediately after the reaction was stopped.

### Screening for genetic material of *Anaplasma* spp., *Ehrlichia* spp., *Borrelia* spp., *Rickettsia* spp. and *Leptospira* spp

Spleen tissue was digested for 6 h with Proteinase K (A&A Biotechnology, Gdańsk, Poland). Extraction of DNA was performed from 10 mg of digested spleen tissue using a Genomic Mini kit (A&A Biotechnology) according to the manufacturer’s instructions. After extractions, all DNA samples were labelled and amplified by real-time PCR using a Rotor-Gene thermocycler (Corbett Research, Mortlake, Australia). The list of primers used for all studied pathogens and the reaction conditions are presented in Table 3. The real-time PCR with SYBR Green 1 dye was carried out in thin-walled test tubes with a capacity of 100 μL. A DyNAmo HS SYBR Green qPCR Kit (Finnzymes, Espoo, Finland) was used to conduct a high-specificity reaction. The reaction mixture with a volume of 20 μL consisted of the following components: 2 μL of the DNA matrix, 0.4 μL of each primer, 10 μL of Master Mix containing a hot start version of the modified Tbr (*Thermus brockianus*) polymerase, buffer for the Tbr polymerase, deoxynucleoside triphosphate, MgCl2, the intercalating SYBR Green 1 dye and water to 20 μL. The obtained PCR products were purified using QIAquick spin columns (Qiagen, Hilden, Germany) and eluted in 50 mL of 10 mM Tris at pH 7.6. The DNA sequence was determined on both strands using the same primers employed for PCR at a DNA sequencing core facility (Institute of Biochemistry and Biophysics, Polish Academy of Sciences, Warsaw, Poland). The sequences were assembled and edited using SeqMan (DNAstar, Lasergene, Madison, WI, USA) and Clustal V alignments.

**Table 3.** Primers and PCR conditions for detection and identification of *Anaplasma/Ehrlichia* spp., *Babesia* spp., *Borrelia burgdorferi* and *Leptospira* spp. in spleen tissue samples from hedgehogs

Pathogen	Primers	Target gene	Amplicon size (base pairs)	PCR conditions	Reference
*Ehrlichia* spp/*Anaplasma* spp.	1: 5′-CTG GGG ACT ACG GTC GCA AGA C-3′ 2: 5′-CTC CAG TTT ATC ACT GGA AGT T-3′	16S–23S RNA	299	×1: den. at 95°C for 600 s ×45: den. at 95°C for 60 s, ann. at 60°C for 60 s, ext. at 72°C for 30 s	6
*Rickettsia* spp.	120-2113: 5′-CGATGCTAACGTAGGTTCTT-3′ 120AA-2235: 5′-CCGGCTATACCGCCTGTAG-3′	rOmpB	773	×1: den. at 95°C for 180 s ×40: den. at 95°C for 30 s, ann. at 50°C for 30 s, ext. at 68°C for 90 s ×1: 68° for 420 s	30
*Borrelia burgdorferi* sensu lato	M1: 5′-ACG ATG CAC ACT TGG TGT TAA-3′ M2: 5′-TCC GAC TTA TCA CCG GCA GTC A-3′	16S RNA	357	×1: den. at 85°C for 600 s ×30: den. at 85°C for 30 s, ann. at 50°C for 30 s, ext. at 65°C for 60 s ×1: ext. at 65°C for 600 s	20
*Leptospira* spp.	G1: 5′-CTG AAT CGC TGT ATA AAA GT-3′ G2: 5′-GGA AAA CAA ATG GTC GGA AG-3′	16S RNA	298–320	×1: den. at 94°C for 180 s ×28: den. at 94°C for 30 s, ann. at 51°C for 60 s, ext. at 72°C for 180 s ×1: ext. at 72°C for 300 s	25

1 den. – denaturation; ann. – annealing; ext. – extension

### Statistical analysis

The analyses were performed using R Studio version 4.1.2 (29), except for prevalence with 95% confidence intervals (CIs), which was determined using an online program (https://epitools.ausvet.com.au/ciproportion). Confidence intervals for prevalence were calculated using the Wilsons core method. Pearson’s chi-square (*χ*^2^) tests were used to analyse the data in different health statuses, sexes and body weights. A P-value of <0.05 was statistically significant.

## Results

No antibodies were found in the tested serum, resulting in a seroprevalence of 0% (0/43; 95% CI: 0.00–8.20) for *C. burnetii*, HEV-3, *T. gondii* and *Trichinella* spp. The genetic material of *Anaplasma* spp. was found in 69.23% of spleens (18/26; 95% CI: 50.01–83.50) and that of *Rickettsia* spp. was noted in 15.38% (4/26; 95% CI: 6.15–33.53). Sequencing of the 299-base-pair product of *Anaplasma* spp revealed the highest (>99%) homology with the 16S–23S intergenic sequence of multiple isolates of *A. phagocytophilum* (GenBank accession Nos U10873, U02521, M73223 and M73224). Sequencing of the 773-base-pair product of *Rickettsia* spp. revealed the highest (>99%) homology with the rOmpB sequence of isolates of *R. helvetica* (GenBank accession Nos MG242260 and GU3244464).

All the *Rickettsia*-positive spleen samples were simultaneously *Anaplasma* positive. *Borrelia* spp., *Ehrlichia* spp. and *Leptospira* spp. genetic materials were not found in this study (0%; 0/26; 95% CI: 0.00– 12.87). Detailed information on *Anaplasma* spp. and *Rickettsia* spp. prevalence among groups of hedgehog samples submitted to PCR are presented in Table 2. A weak correlation between health status and *Anaplasma* spp. positivity was found among different subgroups of hedgehogs (P-value = 0.046, *χ*^2^ = 8.005). Most *Anaplasma* spp.-positive spleen samples originated from hedgehogs with polytrauma, which was most often due to having been struck by a road vehicle (66.67%, 95% CI: 41.71–84.82).

## Discussion

Our study confirmed the presence of *Anaplasma* spp. and *Rickettsia* spp. genetic material in wild hedgehogs from central-western Poland. Because wild hedgehogs are heavily infested with ticks, they are highly exposed to various tick-borne pathogens that may have zoonotic potential, besides being detrimental to the host’s health. Several studies demonstrated that hedgehogs serve as animal hosts for many ticks, including *Ixodes ricinus, Ixodes hexagonus* and *Rhipicephalus sanguineus* (4, 21, 35). These tick species can transmit various pathogens, including zoonotic ones such as *Borrelia* spp., *Rickettsia helvetica* or *Anaplasma phagocytophilum* (21).

*Anaplasma* spp. are the aetiological agents of anaplasmosis, a disease which significantly impacts the health of several animal species and humans and causes economic losses in livestock farming systems (5). Traditionally, the *Anaplasma* genus includes pathogenic *A. phagocytophilum, A. bovis, A. ovis, A. platys, A. marginale* and *A. centrale*, of which the first four species can infect both humans and animals and the remaining two are of veterinary importance, according to published evidence (5). Several studies have confirmed the presence of *A. phagocytophilum* in ticks in Poland. In 2014, Kiewra *et al*. (18) examined 2,507 host-seeking ticks collected in south-western Poland which had been identified as *I. ricinus*, screening them for the presence of *A. phagocytophilum* DNA. The average infection rate reached 4.3% (18). In a recent study conducted in eastern Poland, the DNA of *A. phagocytophilum* was found in 1.28% of 626 examined *I. ricinus* ticks (33). *Anaplasma phagocytophilum* was previously noted in 31.61% (55/174) of the spleen samples obtained from wild carnivores (*Nyctereutes procyonoides, Meles meles, Vulpes vulpes, Martes* sp. and *Mustela putorius*) (39). The presence of *A. phagocytophilum* was also confirmed in the liver and spleen samples from farmed and wild cervids in Poland (*Cervus elaphus, Capreolus capreolus, Dama dama* and *Alces alces*). Out of 207 examined cervids (165 wild and 42 farmed), infection with *Anaplasma* spp. was evident in 43.9% of individuals (91/207). Both spleen and liver samples were obtained from 172 animals, and 81 of them tested positive in at least one sample. However, only 23 animals out of those 81 were positive in both samples. *Anaplasma phagocytophilum* was significantly more often isolated from the spleen than from the liver (66/172 *vs* 38/172) (24).

These results indicate this pathogen’s presence in the environment where European hedgehogs live; however, the pathogen has never been detected in hedgehogs from Poland before. The prevalence of various pathogens in hedgehogs differs geographically. However, *A. phagocytophilum* is the most frequent one reported in studies of these animals (31). The prevalence *of A. phagocytophilum* in our study was high, reaching 69.23% (18/26). Similar results were obtained in Germany, where 61.8% of blood samples (136/220) from European hedgehogs were positive for *A. phagocytophilum* DNA. These samples were from 41 individuals (34). *Anaplasma phagocytophilum* was also found in 76.1% (67/88) of ear tissue samples collected from white-breasted hedgehogs (*Erinaceus roumanicus*) from urban areas in Hungary (11). It was also detected in hedgehogs in the Czech Republic. In the study by Lesiczka *et al*. (21), ear, muscle, lung, liver, spleen, urinary bladder, kidney, brain and blood tissue samples were collected from hedgehog carcasses. Blood and skin samples were also collected from live-trapped hedgehogs, and both live-animal and carcass samples were tested for the presence of *A. phagocytophilum* DNA by quantitative PCR. An individual animal was considered positive when at least one of its tissue samples was positive. The positivity rate was high in both *E. europaeus* and *E. roumanicus*: the former species’ carcasses were 97.6% positive and 93.1% of live specimens carried the bacterium’s genetic material, and the latter species’ rates were 97.6% and 85.7%, respectively (21). In a study conducted in the Netherlands by Krawczyk *et al*. (19), *A. phagocythophilum* was found in 74/277 (27%) *I. hexagonus* and 6/25 (24%) *I. ricinus* ticks collected from live hedgehogs. The pathogen is not only reported in Europe. In eastern China, 43.8% (14/32) of Amur hedgehogs (*E. amurensis*) were *Anaplasma* spp. positive. The researchers in that region found the pathogen in different organs: in 25% (8/32) of spleen samples, 25% (8/32) of lung samples, 18.8% (6/32) of liver samples, 15.6% (5/32) of heart samples, 12.5% (4/32) of intestine samples and 9.4% (3/32) of kidney samples (31). In central China, 26% (19/73) of blood samples from Amur hedgehogs were positive (40). In Iran, the presence of *A. marginale* infection was confirmed in 3.8 % (2/53) of the blood samples from long-eared hedgehogs (*Hemiechinus auritus*) and confirmed in *Rhipicephalus turanicus* ticks collected from them (17). Conversely, Sarani *et al*. (32) did not detect the genetic material of *Anaplasma* spp. in blood samples (0/51) of long-eared hedgehogs in the same country. Orkun *et al*. (26) examined ticks collected from hedgehogs (*Erinaceus concolor*) in Turkey, but the investigation did not reveal *Anaplasma* spp. DNA. Balti *et al*. (2) also did not find *Anaplasma* spp. in Algerian hedgehogs from Tunisia (0/20) when examining blood, kidney, spleen and bone marrow fluid samples. In our research, we noted a potential link between hedgehogs diagnosed with polytrauma and positivity for *Anaplasma* spp. (P-value = 0.045). The symptoms of anaplasmosis in hedgehogs are not extensively documented. However, they may resemble those observed in other animals. Nonspecific symptoms such as anorexia, fever, lethargy, weakness, anaemia, eye and oral discharge, skin abnormalities and respiratory distress have been noted in dogs and cats (1). The observed correlation of polytrauma and *Anaplasma* spp. infection could be attributed to the overall debilitation of the hedgehogs making them more vulnerable to predator attacks and impairing their ability to sense and evade road vehicles.

The *Rickettsia* genus in the *Rickettsiaceae* family comprises obligate intracellular pathogens capable of infecting humans and animals with mild-to-severe symptoms. *Rickettsiae* are Gram-negative bacteria divided into two main groups: those causing typhus and those causing spotted fever (12). The prevalence of *Rickettsia* species in our study reached 15.38% (4/26). Previously, rickettsial DNA was detected in at least 279 of 1,148 ticks (*I. ricinus* and *D. reticularis*) collected in north-eastern Poland. Three species were identified in that study: *R. helvetica, Candidatus R. mendelii* and *R. raoultii* (36). *Rickettsia helvetica* has been also identified in ticks obtained from *Lacerta agilis* and *Zootoca vivipara* lizards in urban regions of southwestern Poland, having been evident in 19.3% of 445 specimens (9). In 2009, *R. helvetica* was detected in 8% (1/12) of nymphs and in at least 10.7% of 804 larvae of *I. ricinus* collected from rodents (*Apodemus flavicollis* and *Myodes glareolus*) in central-western Poland; however, none of the blood samples collected from the 13 rodents studied was positive for *Rickettsia* spp. (7). Similarly, in a study conducted earlier in the same region of Poland, none of the 323 blood samples collected from wildlife (130 birds, 149 rodents and 44 cervids) was PCR positive for *Rickettsia* spp. (37). However, Gajda *et al*. (13) examined 193 spleen samples and 144 blood samples obtained from 193 wild-living *Apodemus agrarius, Apodemus flavicollis* and *Myodes glareolus* and found 17.6% (34/193) of the spleen samples positive for *Rickettsia* spp. The blood samples were markedly less frequently positive: *Rickettsia* spp. were confirmed in only four, in two of which the identified pathogen was *R. helvetica*. These results may be due to the *Rickettsia* DNA presence in blood likely being sparse and short-lived in acute infections (38). To the best of the authors’ knowledge, our research represents the first identification of *R. helvetica* in wild hedgehogs in Poland. Comparable outcomes have been reported by researchers in other countries. For example, Balti *et al*. (2) found the DNA of *Rickettsia* spp. in 10% of tested samples from Algerian hedgehogs (*Atelerix algirus*) in Tunisia. In China, 17.8% (8/45) of hedgehogs yielded samples of muscle, heart, liver, spleen, lung, kidney, brain or intestine tissue which were PCR positive for *Rickettsia* spp. (41). However, in another study conducted in China, no DNA of these bacteria was found in hedgehogs (0/73) (40). There are few studies on the detection of *Rickettsia* spp. in hedgehogs themselves. Nevertheless, the pathogen is also detected in ticks parasitising hedgehogs, suggesting that hedgehogs are exposed to *Rickettsia* spp. infection. Orkun *et al*. (26) found a high prevalence of *Rickettsia* spp. in by-group pooled samples of ticks collected from 12 hedgehogs in Turkey (65.8%, 27/41). Barradas *et al*. (4), who conducted a study in Portugal, found *Rickettsia* DNA in 10.38% (22/212) samples from *Rhipicephalus sanguineus*. None of the *I. hexagonus* tested positive for *Rickettsiae* (0/48). *Rickettsia asembonensis* DNA was also identified in 47% (55/117) of *Archaeopsylla erinacei* (hedgehog fleas) tested (4).

Our study demonstrated that all four spleens positive for *Rickettsia helvetica* were also positive for *Anaplasma phagocytophilum*. Coinfections with multiple pathogens were observed to be in the majority in both ticks and their hosts. In the study of Qi *et al*. (28), the authors investigated coinfections of tickborne pathogens in ticks and their wild hedgehog hosts in Jiangsu province, eastern China. A 74.9% proportion (131/175) of *Haemaphysalis flava* ticks was coinfected with more than one pathogen. Dual coinfections with *Coxiella* spp. and *Rickettsia* spp. predominated (43.4%), and triple coinfections with *Coxiella* spp., *Ehrlichia* spp. and *Rickettsia* spp. were also frequent. In hedgehogs, dual pathogen coinfections with *Coxiella* spp. and *Ehrlichia* spp. and triple pathogen coinfections with *Anaplasma* spp., *Coxiella* spp. and *Ehrlichia* spp. were most heavily represented with rates of 40% and 30%, respectively (28). Similar observations on coinfections were reported in ticks, fleas and their hedgehog hosts in Tunisia (2). Coinfections in hedgehogs may result from cross-infections between parasites and hosts during blood feeding.

The genetic material of *Borrelia* spp., *Ehrlichia* spp. and *Leptospira* spp. was not found in our study. However, there are reports worldwide confirming the occurrence of these pathogens in hedgehogs (2, 26, 28, 32, 35, 40). None of the hedgehogs in our study were positive for antibodies against *C. burnetii*, HEV-3, *T. gondii* or *Trichinella* spp., suggesting they were not exposed to these pathogens. However, infection of hedgehogs with *Coxiella* and *T. gondii*, and the presence of genetic material of these pathogens in hedgehog tissues were reported by other authors (10, 28). Hosni *et al*. (16) detected *Trichinella spp*. larvae in abdominal, hindlimb and forelimb muscle and whole diaphragm samples from hedgehogs. Hepatitis E virus genotype 3 (also known as *Paslahepevirus balayani* genotype 3) has not been described in hedgehogs before. It is reported to infect small mammals that hedgehogs may feed on, which puts them at risk of becoming infected. The RNA of HEV-3 was detected for the first time in a liver sample (0.21%; 1/483) originating from a yellow-necked mouse (*Apodemus flavicollis*) collected in 2014 in Croatia (27), which is a possible route of infection of the hedgehog in the present study because the yellow-necked mouse is a synanthropic species inhabiting Poland’s cities (22).

It is essential to be aware that our research has limitations. Further investigation into more hedgehogs is necessary, considering the relatively small size of the sample in the present study. Additionally, only spleen samples underwent PCR testing. In infected hedgehogs, the prevalence of *Anaplasma* spp. or *Rickettsia* spp. may vary in different organs. Such a variation was observed in the study by Qi *et al*. (28) regarding *Anaplasma* spp.; however, in general, they noted higher pathogen rates in the hedgehogs’ spleens, livers, and lungs. These differences in bacterial distribution between anatomical sites may be attributed to individual variations, including immune status, overall pathogen loads and the co-existence of other bacteria. Serological methods detecting antibodies (*e.g*. by ELISA) are a better option for screening the population for the presence of the pathogens in question. Unfortunately, no multispecies serological tests suitable for reliable detection of antibodies against *Anaplasma* spp., *Rickettsia* spp., *Borrelia* spp., *Ehrlichia* spp. and *Leptospira* spp. in the serum of hedgehogs are currently available. Therefore, in the present study, we decided to use the PCR method for screening for pathogens in hedgehogs. However, instead of conducting this screening on blood samples, we chose spleen tissue as our target. This decision was informed by the significantly reduced risk of encountering false-negative results in PCR tests performed on spleen tissue, in comparison to the risk when conducting them on blood samples. Chain-reacting haematological samples can often lead to such results where bacteraemia, spirochetaemia or rickettsaemia are absent.

## Conclusion

The present study is the first comprehensive investigation into the prevalence of zoonotic pathogens in wild hedgehogs from Poland. Our findings suggest that hedgehogs may serve as reservoirs of *Anaplasma phagocytophilum* and *Rickettsia helvetica*. Hedgehogs’ exact role in transmitting tick-borne infections remains unclear. As the hedgehog population grows and ticks become more prevalent in human environments, further research in this field becomes crucial. It is important to assess the extent of hedgehog involvement in these pathogens’ enzootic cycle and better understand the transmission mechanisms and cycles involving hedgehogs and ticks. Our results indicate that as a synurbic species, hedgehogs could contribute to spreading and transmitting tick-borne pathogens in urban areas, posing potential public health risks. The interaction between humans and urban wildlife establishes opportunities for cross-species transmissions and new outbreaks of zoonotic diseases, and as these interactions rise in frequency, so the need grows to address this issue through the One Health approach. Consequently, incorporating hedgehog research into the One Health approach is essential for it to maintain effectiveness in addressing health and ecological dynamics as human settlements and animal habitats increasingly overlap.
